# Phylogenomic Analysis of Secondary Metabolism in the Toxic Cyanobacterial Genera *Anabaena*, *Dolichospermum* and *Aphanizomenon*

**DOI:** 10.3390/toxins12040248

**Published:** 2020-04-11

**Authors:** Julia Österholm, Rafael V. Popin, David P. Fewer, Kaarina Sivonen

**Affiliations:** Department of Microbiology, University of Helsinki, Viikinkaari 9, FI-00014 Helsinki, Finland; julia.osterholm@helsinki.fi (J.Ö.); rafael.popin@helsinki.fi (R.V.P.); david.fewer@helsinki.fi (D.P.F.)

**Keywords:** cyanobacteria, biosynthetic gene cluster, cyanotoxin, microcystin, anatoxin, natural products, phylogenomics

## Abstract

Cyanobacteria produce an array of toxins that pose serious health risks to humans and animals. The closely related diazotrophic genera, *Anabaena*, *Dolichospermum* and *Aphanizomenon*, frequently form poisonous blooms in lakes and brackish waters around the world. These genera form a complex now termed the *Anabaena*, *Dolichospermum* and *Aphanizomenon* (ADA) clade and produce a greater array of toxins than any other cyanobacteria group. However, taxonomic confusion masks the distribution of toxin biosynthetic pathways in cyanobacteria. Here we obtained 11 new draft genomes to improve the understanding of toxin production in these genera. Comparison of secondary metabolite pathways in all available 31 genomes for these three genera suggests that the ability to produce microcystin, anatoxin-a, and saxitoxin is associated with specific subgroups. Each toxin gene cluster was concentrated or even limited to a certain subgroup within the ADA clade. Our results indicate that members of the ADA clade encode a variety of secondary metabolites following the phylogenetic clustering of constituent species. The newly sequenced members of the ADA clade show that phylogenetic separation of planktonic *Dolichospermum* and benthic *Anabaena* is not complete. This underscores the importance of taxonomic revision of *Anabaena*, *Dolichospermum* and *Aphanizomenon* genera to reflect current phylogenomic understanding.

## 1. Introduction

Cyanobacteria are photosynthetic bacteria that frequently form toxic blooms in lakes and brackish waters. Toxic strains cause animal poisoning [1,2] and pose a health threat to humans, thus preventing recreational and drinking use. The frequency and intensity of toxic blooms have increased in recent decades and there is growing evidence that global climate warming favors this development [3,4].

The *Anabaena*, *Aphanizomenon* and *Dolichospermum* genera form a phylogenetically closely related genera complex (ADA clade) of filamentous, nitrogen-fixing cyanobacteria, which is known for the production of an array of toxins [5,6]. Cyanobacteria assigned to the genus *Anabaena* have been reported to produce more different toxins than any other cyanobacteria group, including hepatotoxic microcystin [7,8], cytotoxic cylindrospermopsin [9], neurotoxic anatoxin-a [10], guanitoxin [11,12], and saxitoxin [13,14]. Some *Dolichospermum* and *Aphanizomenon* strains also produce geosmin, a volatile odorous metabolite, which frequently causes taste and odor problems in drinking water production [5,6].

The problematic nature of cyanobacterial taxonomy is emphasized by the rapidly emerging body of genome sequence data and it is clear that traditional taxonomy based on morphological features does not accurately describe the phylogenetic relationships between cyanobacteria [15]. The genus *Anabaena* consists of morphologically similar strains occupying different habitats. Planktonic *Anabaena* produce gas vesicles that enable their buoyant lifestyle and these strains can form blooms, strains without gas vesicles have benthic or soil inhabiting lifestyle [16,17]. Benthic and planktonic strains of *Anabaena* were shown to constitute phylogenetically separate entities a decade ago despite their morphological similarity and planktonic strains were reclassified as a new genus, *Dolichospermum* [17]. *Aphanizomenon* is morphologically distinct from *Anabaena* and *Dolichospermum* [18], but all these three genera are intermixed phylogenetically in what has been called the ADA clade [6,19]. *Anabaena* and *Aphanizomenon* strains are also found outside the ADA clade, and some of these strains have been reassigned to other genera, for instance *Sphaerospermum* and *Chrysosporum* [20,21]. Both new and old names are currently used interchangeably, which adds to taxonomic confusion.

Cyanotoxins and other natural products are synthesized on enzymatic assembly lines that are encoded by biosynthetic gene clusters (BGCs) [22]. BGCs can be identified from genomes using known chemical structures as a guide, but some BGCs have been discovered through genome mining [23]. Although cyanobacterial natural products are studied intensively, the obscurity of nomenclature and taxonomy of these organisms makes it difficult to determine the genuine distribution of bioactive compounds in the taxa [23]. Two recent studies on 15 [6] and 17 ADA genomes [19] revealed an array of natural product BGCs and established four phylogenetic subgroups within the clade. These subgroups were proposed to represent four species in which toxin BGCs were sporadically present [6]. However, the number of genomes was insufficient to determine which traits would define these species and these subgroups were not consistent with division to *Anabaena*, *Dolichospermum* and *Aphanizomenon* genera. These studies focused on toxic bloom-forming strains, rather than benthic strains, which are over-represented in culture collections and genome databases. However, the genus *Anabaena* is defined as benthic and a wider understanding of taxonomy and natural product distribution of this genera complex would benefit from the inclusion of more genetic diversity. 

The aim of our study was to expand the genomic coverage of the ADA clade. We analyzed 20 publicly available and 11 newly sequenced genomes from diverse habitats for natural product BGCs to study the frequency and distribution of natural product biosynthetic pathways. We discovered a correlation between phylogeny and natural product capability with toxin BGCs being more frequent or even limited to a certain species-like clusters within the ADA clade. We also demonstrate that all previously available *Anabaena* genomes in the ADA clade encode gas vesicle gene clusters and should be transferred to the genus *Dolichospermum*. 

## 2. Results

### 2.1. Phylogeny of the ADA Clade

**Figure 1:**
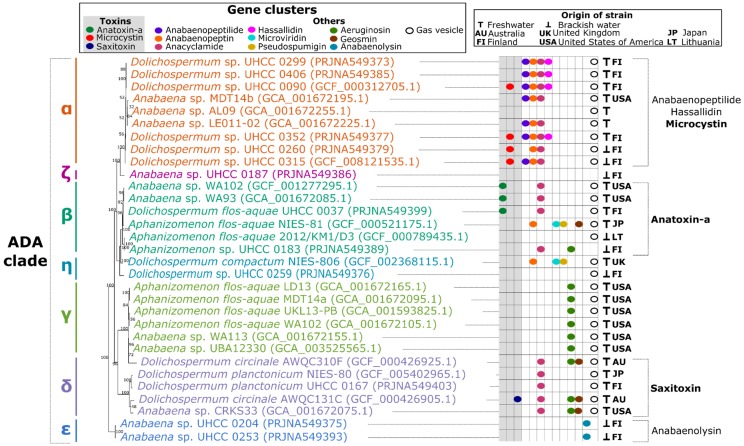
Fragment of a maximum likelihood tree constructed using 31 universal marker genes [24,25] encoded in 31 *Anabaena/Dolichospermum/Aphanizomenon* genomes. The proposed *Anabaena/Dolichospermum/Aphanizomenon* (ADA) subgroups [6,19] and natural products biosynthetic gene clusters are indicated by different colors. Genome accession numbers in NCBI are presented within parentheses. For the complete tree that includes 94 genomes from diverse cyanobacterial families, see Figure S2.

We obtained 11 new draft genome sequences for 11 strains belonging to the ADA clade, isolated from planktonic and benthic, toxic and non-toxic, freshwater and brackish water samples in Finland. These were combined with 20 previously available ADA genomes and used for phylogenomic analysis (Table S1). A maximum likelihood tree constructed using 31 universal marker genes shows that the 31 genomes were divided to 7 distinct ADA subgroups (Figure 1). These subgroups were given the names ADA-α to ADA-η. To determine how representative these 31 genomes were of the ADA clade, a Bayesian tree based on the 16S rRNA gene was constructed and average nucleotide and amino acid identity were calculated to generate heatmaps (Figure S1). The results corroborate the ADA clade subgroups classification achieved in the 31 marker proteins tree (Figure 1). Most strains belong to the same subgroup in the 16S rRNA tree when compared with the 31 proteins tree. One exception is the strain *Dolichospermum circinale* AWQC310F, which clusters with ADA-δ in the 16S tree but with ADA-γ in the 31 proteins tree.

### 2.2. Distribution of Toxin Biosynthetic Pathways 

The 31 genomes were analyzed for the presence of natural product BGCs and gas vesicle gene clusters (Figures S3 and S4). The distribution of these gene clusters was compared to the phylogenomic 31 protein tree (Figure 1). This comparison revealed a correlation between the distribution of the gene clusters and the subgroup division. Microcystin, anatoxin-a, and saxitoxin BGCs were limited to subgroups ADA-α, ADA-β, and ADA-δ, respectively (Figure 1). Cylindrospermopsin BGC was not found in any of the genomes. The only strain with toxin BGC, which lacked prior evidence for corresponding toxin production was *Dolichospermum* sp. UHCC 0352 and for that microcystin production was verified with LC-MS (Table S1). 

We also mapped the distribution of strains known to produce microcystin, anatoxin-a, saxitoxin, and cylindrospermopsin to a 16S rRNA phylogenetic tree (Figure S2). This phylogenetic tree also revealed a similar distribution of toxin BGCs (Figure 1). The microcystin BGC was found only in ADA-α and microcystin-producing strains *Anabaena* spp. BIR250A, BIR257, and BIR258 from the Baltic Sea belong to this subgroup based on 16S rRNA sequence [7]. However, BIR246, also a microcystin producer, is in a branch of its own within the ADA clade (Figure S1) [7]. Anatoxin-a BGCs and producers were limited to ADA-β. Cylindrospermopsin producers *Aphanizomenon* spp. 10E3, 10E9, 22D11, and 30D11 were also placed in ADA-β in the 16S rRNA tree (Figure S1) [9]. Even though these two toxins were both found in ADA-β, they were not detected simultaneously in a single strain. The saxitoxin BGC was found only in the genome of *D. circinale* AWQC131C, which belongs to ADA-δ.

### 2.3. Distribution of Other Natural Products and Gas Vesicle Gene Clusters

We screened other natural product BGCs and in some cases the distribution was concentrated to certain subgroups as observed for toxin BGCs. Anabaenolysin was limited to subgroup ADA-ε, whereas anabaenopeptilide and hassallidin BGCs were found only in ADA-α. By contrast, aeruginosin, anacyclamide, anabaenopeptin, geosmin, microviridin, and pseudospumigin BGCs had a wider distribution and were found in two or more subgroups (Figure 1).

The gas vesicle gene cluster was found in the majority of strains but was absent from the benthic *Anabaena* strains UHCC 0187, 0204, and 0253 and *Aphanizomenon* UHCC 0183 (Figure 1, Table S2, Figure S4). Although the *Aphanizomenon flos-aquae* UKL13-PB genome lacks annotation in NCBI, protein BLAST recognized nine adjacent gas vesicle genes.

#### 2.3.1. ADA-α, the Microcystin Producers

*Dolichospermum* spp. UHCC 0260, 0299, 0352, and 0406 were positioned in the subgroup with previously published *Dolichospermum* spp. UHCC 0090 and 0315 and three other *Anabaena* genomes (Figure 1). Microcystin, hassallidin, and anabaenopeptilide BGCs were found only in genomes from this subgroup. Apart from strain *Anabaena* sp. MDT14b, all others have at least three known BGCs. Genomes encoding the microcystin BGC are all planktonic *Dolichospermum* from Finland and members of this subgroup have the gas vesicle gene cluster. 

#### 2.3.2. ADA-β, the Anatoxin-a and Cylindrospermopsin Producers, and Its Sister Subgroup ADA-η

The subgroup β included *Aphanizomenon* sp. UHCC 0183 and *Dolichospermum flos-aquae* UHCC 0037 in addition to five previously published *Anabaena*, *Aphanizomenon* and *Dolichospermum* strains. The anatoxin-a BGC is restricted to this subgroup (Figure 1). Anatoxin-a producing *Dolichospermum* (previously *Anabaena*) sp. 86 also belong to subgroup β [26] (Figure S1). The only strain lacking the gas vesicle gene cluster is *Aphanizomenon* sp. UHCC 0183. ADA-β is the most diverse among the subgroups. It has the widest range of different BGCs, members of this subgroup carry the name of all three genera, and it is geographically and environmentally dispersed; strains were isolated from Finland, USA, Japan, and Lithuania and from both freshwater and brackish water environments (Table S1). Genomes with anatoxin-a originate from freshwater lakes and also have anacyclamide BGC but no other known BGCs. Cylindrospermopsin BGC was not found in any of the genomes but the 16S rRNA sequence tree placed cylindrospermopsin-producing *Aphanizomenon* spp. 10E3, 10E9, 22D11, and 30D11 in this group (Figure S1) [9].

*Dolichospermum* sp. UHCC 0259 and *Dolichospermum compactum* NIES-806 formed the new subgroup ADA-η based on the 16S tree and identity heatmaps (Figure S1). This group is closely related to ADA-β and an oligopeptide gene island formed by pseudospumigin, anabaenopeptin, and microviridin BGCs was found only in both of these subgroups. Both strains in this group contain the gas vesicle gene cluster.

#### 2.3.3. ADA-δ, the Saxitoxin Producers, and Its Sister Subgroup ADA-γ

*Dolichospermum planctonicum* UHCC 0167 was a new addition to subgroup ADA-δ, which was the only one to present the saxitoxin BGC (Figure 1). Common features of this freshwater group were anacyclamide BGC and gas vesicle gene clusters. Geosmin BGCs were also concentrated in this group, as three out of four geosmin BGCs in our dataset were found here. Aeruginosin BGC was found in three genomes in ADA-δ.

The subgroup γ, which remained unaltered as a sister clade of δ, showed genomes with aeruginosin and no other known BGCs. The strains have gas vesicle clusters and originate from USA. Four of them are *Aphanizomenon flos-aquae* and two are *Anabaena* from metagenomic assemblies.

#### 2.3.4. ADA-ε and ADA-ζ, the Benthic Subgroups

*Anabaena* spp. UHCC 0204 and UHCC 0253 formed the new subgroup ADA-ε (Figure 1). This subgroup was the only one to include genomes encoding the anabaenolysin BGC. Both strains originate from brackish water in the Baltic Sea and are benthic and lack a gas vesicle gene cluster. *Anabaena* sp. UHCC 0187 represents the only member of the subgroup ζ. The strain is benthic and was isolated from brackish water in the Baltic Sea and encodes no known BGCs or gas vesicle genes.

### 2.4. Natural Products Biosynthesis Networks

**Figure 2:**
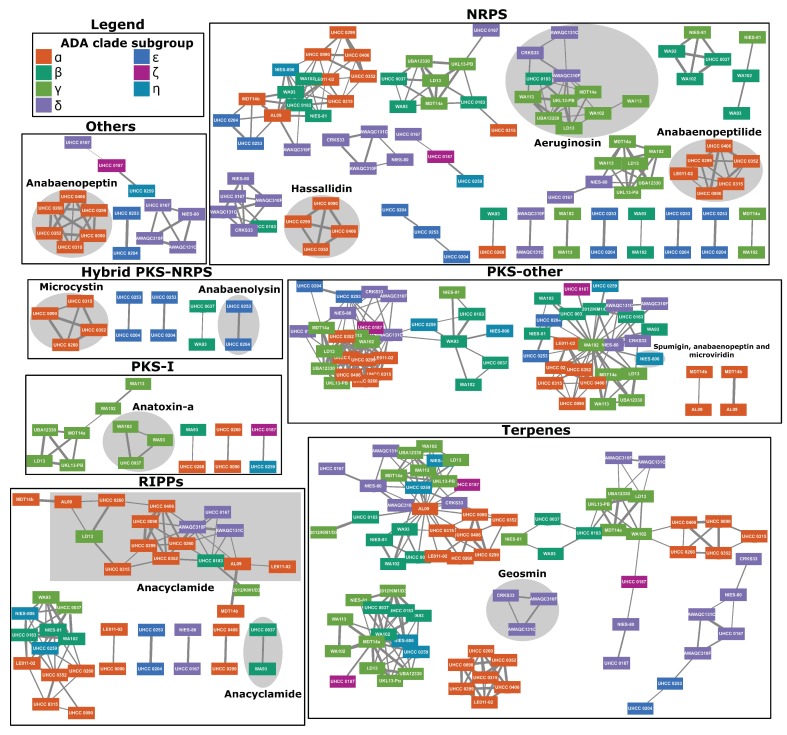
Network of automatically annotated and organized regions with biosynthetic gene clusters from the 31 analyzed *Anabaena/Dolichospermum/Aphanizomenon* genomes. Singletons are not represented in the figure. Known natural product BGCs are marked with grey background. Automatic organization of regions is not fully congruent with manual classification of BGCs.

Automatic prediction identified 359 natural product BGCs in the 31 genomes. These ranged from 8 to 16 BGCs per genome and most had unknown products. These BGCs were classified into seven classes, with the NRPS pathways being the most widespread antiSMASH recognized pathways in the genomes (102 BGCs), followed by Terpenes (83 BGCs), PKS-other (55 BGCs), RiPPs (50 BGCs), Others (34 BGCs), PKS-I (16 BGCs), and hybrid PKS-NRPS (19 BGCs) (Figure 2). Manual curation of the regions revealed the presence of 11 known natural product BGCs in the 31 ADA clade genomes (Figure S2, Table S2). The products of these natural product BGCs belonged to the following different biosynthetic classes: alkaloid (saxitoxin), alkaloid/PKS (anatoxin-a), PKS-NRPS (anabaenolysin, microcystin, spumigin), NRPS (aeruginosin, anabaenopeptilide, anabaenopeptin, hassallidin), RiPPs (anacyclamide, microviridin), and terpene (geosmin). With the exception of anacyclamide, the other natural product BGCs were relatively conserved in the analyzed strains, showing differences mainly in gene sizes and in some cases the presence or absence of tailoring enzymes (Figure S3). The anacylamide BGC was present in the majority of the strains but was more variable.

The BGC regions were used for generating a network of clusters with more than one representative (Figure 2). In total, 51 groups were formed in the network, of which 20% were associated with known natural products and 80% with unknown products. The genomic regions encoding hassallidin and anabaenopeptilide BGCs, mainly from *Dolichospermum* strains from Finland, were categorized in the NRPS class. The aeruginosin BGCs were identified mainly in strains from the USA and Australia. The terpene class consisted of geosmin regions from Australian and USA strains. In contrast, the one from NIES-81 was a singleton and therefore is not shown in the figure. Even though spumigin, anabaenopeptin, and microvirin BGCs contain NRPS and PKS genes, the genomic regions were classified in the PKS-other class. The strain NIES-81 encodes similar BGCs, but the region did not cluster with any other and is therefore not represented in the figure. The RiPPs class contained the anacyclamide BGCs that are present in several *Anabaena* and *Dolichospermum* strains from Australia, Finland, and unknown origins. Anabaenopeptin, which was identified only in Finnish *Dolichospermum*, was classified in others. The class PKS-I consisted of anatoxin-a BGCs from Finland and USA. Finally, Hybrid PKS-NRPS regions included microcystin from *Dolichospermum* and anabaenolysin from *Anabaena*, all from Finland. The saxitoxin gene cluster from *Dolichospermum circinale* AWQC131C was also a singleton and is not present in the network figure.

## 3. Discussion

### 3.1. Distribution of Toxins and Other Natural Products Follows Phylogenetic Clustering in ADA Clade 

We compared the genomes of 11 newly sequenced and 20 previously available *Anabaena/Dolichospermum/Aphanizomenon* strains that cluster in distinct subgroups of the ADA clade. We found that distribution of toxin and other natural product BGCs follow phylogenomic clustering. Each toxin BGC was found only in one subgroup. Nevertheless, toxic and non-toxic strains were intermixed as has been shown earlier for microcystin [7] and cylindrospermopsin [9]. None of the toxins are produced exclusively by members of the ADA clade. Microcystin, cylindrospermopsin, anatoxin-a, and saxitoxin are all produced by several cyanobacteria genera, including *Microcystis*, *Planktothrix*, *Phormidium*, *Cylindrospermopsis*, *Oscillatoria* and, in the case of saxitoxin, even by some eukaryotic dinoflagellates [27]. 

ADA-α strains with microcystin BGCs, *Dolichospermum* sp. UHCC 0090 [28], UHCC 0260 [7], UHCC 0315 [19], and UHCC 0352 produce microcystin. Neurotoxic strains were located in subgroups β (anatoxin-a) and δ (saxitoxin). Anatoxin-a production has been shown in all three strains containing anatoxin-a BGCs [10,29]. The only saxitoxin-producing strain in our study, *D. circinale* AWQC131C, belongs to the subgroup δ based on the 16S RNA tree [30]. *D. circinale* AWQC310F, which is considered a non-toxic sister strain, was clustered in γ in the 31-protein phylogenomic tree. However, the natural product BGC content of AWQC310F and 16S analyses and heatmaps demonstrate that these strains are more similar to group δ. Cylindrospermopsin BGCs were not found in any of the genomes in our study, but *Aphanizomenon* spp. strains 10E6, 10E9, 30D11, and 22D11, which are known cylindrospermopsin producers [9], cluster in the subgroup β alongside anatoxin-a producing *Dolichospermum* strains. Additional strains of *Aphanizomenon* are reported to produce cylindrospermopsin but these are now assigned to *Chrysosporum* [9,31] or otherwise genera that are clearly outside the ADA clade [32].

ADA strains produce other natural products, in addition to toxins, with a range of bioactivities, including compounds with antimicrobial, protease inhibitory, and anticancer activities [23]. We identified natural product BGCs for anabaenopeptilide, anabaenopeptin, anacyclamide, hassallidin, microviridin, pseudospumigin, aeruginosin, geosmin, and anabaenolysin from the 31 ADA genomes. Some of these natural product BGCs segregated according to phylogenetic subgroups, similar to toxin BGCs. Moreover, there seems to be a segregation of certain BGC combinations. Anabaenopeptilide, anabaenopeptin, and anacyclamide gene clusters are present in seven out of nine members of subgroup α, while aeruginosin, anacyclamide, and geosmin BGCs are in three out of five members in subgroup δ. In ADA strains *D. compactum* NIES-806 and *Aphanizomenon flos-aquae* NIES-81, anabaenopeptin, microviridin, and spumigin clusters are located so closely that they form an oligopeptide island (Figure S3). A similar gene island consisting of anabaenopeptin, microviridin, and cyanopeptolin can also be found in *Planktothrix rubescens* NIVA CYA 98 [33]. An anabaenopeptin-spumigin island can be found in *Nodularia spumigena* CCY9414 and *Sphaerospermopsis torques-reginae* ITEP-024 [34]. In the latter case, enzymes encoded by the *hphABCD* genes in apt cluster are used in the supply of homo-amino acids to both spumigin and anabaenopeptin biosynthesis [34]. HphABCD genes are found also in the spu-apt-mvd gene island of ADA strains NIES-806 and NIES-81 and likely serve the same purpose here. Co-occurrence of the BGCs may also indicate a common target or synergy between the natural products. For example, synergy has been shown for anabaenolysin and cyclodextrins, in which the latter enhances the antifungal activity of the former [35]. 

### 3.2. Genetic Separation into Benthic and Planktonic Branches is Not Complete

The majority of benthic ADA strains in our study belong to subgroup ε, which branch clearly separately from the intermixed planktonic *Dolichospermum*/*Aphanizomenon* branch. However, benthic *Anabaena* sp. UHCC 0187 which is the only member of subgroup ζ clusters closer to planktonic members of ADA clade than the other benthic subgroup ε. This contradicts the previous findings that benthic and planktonic *Anabaena*/*Dolichospermum* strains are genetically distinct [17]. Furthermore, *Aphanizomenon* sp. UHCC 0183 lacks a gas vesicle gene cluster although it belongs to the planktonic branch. *Aphanizomenon* sp. UHCC 0183 was isolated from a planktonic sample and *Aphanizomenon* as a genus is considered planktonic [36]. This strain was isolated in 1993 and may have lost the gas vesicle gene cluster during decades of cultivation in laboratories, as has been reported with *Microcystis aeruginosa* [37]. Transition from planktonic to benthic lifestyle has been reported also in *Dolichospermum* sp. UHCC 0315 in which two substrains were found. One substrain was buoyant with gas vesicles and the other had lost some of the gas vesicle genes and was growing in the bottom of the cultivation bottle [19]. This raises the question of whether similar transition from planktonic to benthic lifestyle could happen also in the nature, and if this has happened to *Anabaena* sp. UHCC 0187. Future studies can possibly present new genomes that will expand the benthic subgroups ε and ζ and help clarify the relationship of benthic and planktonic strains. 

All *Anabaena* strains from which we found gas vesicle gene cluster, namely *Anabaena* spp. WA113, WA102, WA93, UBA12330, MDT14b, LE011-02, CRKS33, and AL09, should be renamed *Dolichospermum* according to the current nomenclature guidelines [17]. Thus, the remainder of the ADA clade (subgroups α, β, γ, δ, and η) would consist of planktonic *Dolichospermum* and *Aphanizomenon*. Clustering into subgroups in the planktonic *Dolichospermum/Aphanizomenon* branch does not clearly follow the morphology-based nomenclature into *Dolichospermum* and *Aphanizomenon*, which is supported by earlier studies [38]. Subgroups ADA-α, ADA-δ, and ADA-η consist of *Dolichospermum* (or *Anabaena* with gas vesicle gene cluster); ADA-β and ADA-γ are a mixture of *Dolichospermum* and *Aphanizomenon.*

When the ADA clade was first introduced, it was suggested to represent one genus and the subgroups were seen as species [6]. The average 16S rRNA gene sequence similarity between benthic *Anabaena* and planktonic *Dolichospermum*/*Aphanizomenon* is 96.7% (Table S3), which is clearly above the suggested threshold of 95% for genus delineation [39]. Therefore, suggesting that the ADA clade represents one genus seems to be reasonable [6], but including the benthic *Anabaena* subgroup ε in this clade is also justified. Average nucleotide identity (ANI) is regarded as a new gold standard for taxonomic delineation as it is less laborious than DNA-DNA hybridization and more reliable than 16S rRNA comparison [40,41]. Although values between 95% and 96% have generally been accepted as a threshold for species delineation [42], even higher boundaries of 96.5% and 98.65% have been suggested [40,43]. In a previous study, ANI values within each ADA subgroup were above the species cutoff score (95.9%–99.29%) [6], but our results indicate 2.23 to 6.42 percentage points lower with the same genome pairs (Table S4). In this case, ANI values in subgroups were on average under the lowest species cutoff of 95% (90.63%–97.15%) in all subgroups other than ADA-γ (95.8%–97.84%; Table S4). Consequently, species delineation in the ADA clade is still not clear and needs further investigation. It has been pointed out before that ANI values differ depending on the calculation tool used and the magnitude of difference depends on the compared genomes [44]. For now, the difference in ANI values in the ADA clade presented by these two methods is so large that the 95% threshold values cannot be used for taxonomic delineation or it would lead to contradictory results depending on the program used.

Although the taxonomy of *Anabaena*-like cyanobacteria has been revised, several *Anabaena* strains clearly cluster outside the ADA clade such that the nomenclature and taxonomy of genus *Anabaena* is not yet fully resolved [6,19]. Some of the previously named planktonic *Anabaena* and *Aphanizomenon* species that do not cluster within the ADA clade have been transferred into genera *Sphaerospermum* and *Chrysosporum* [20,21]. We think that transferring all *Anabaena* strains that cluster outside the ADA clade to different genera can be justified as the type species of *Anabaena*, *Anabaena oscillarioides* [45], fits in the subgroup ADA-ε. Consequently, this group has the priority to the name. Future studies with more genomes will help to clarify the taxonomy of the ADA clade further.

### 3.3. Core Versus Pan Genome

Previous analyses of the ADA clade core genome reported an estimated value of 1500 genes [6,19]. However, the present analysis suggests that the core genome is approximately 1400 genes, or 31% of the average number of genes per genome (4500) (Figure S5). This difference of 100 genes represents approximately 2% of the average and is likely related to the exclusion of genomes outside of the subgroups α to ε and the addition of several new genomes [19]. In comparison, the cyanobacteria core genome size is approximately 559 genes [46], which is less than half the size of the core genome of the ADA clade. The pan genome increased 2900 genes from the 12,000 previously reported [19] and is now larger than those calculated for *Microcystis aeruginosa* (12,000; [47]) and *Raphidiopsis* (4700; [48]) but smaller than *Plankthothrix* (15,000; [49]).

### 3.4. ADA Genomes Encode a Vast Array of BGCs

Manual curation of cyanobacterial genomes has led to the description of many BGCs involved in the synthesis of natural products [34,50,51,52]. Nevertheless, most of the natural product BGCs encoded in these genomic sequences have unknown products [53,54]. The 31 ADA genomes encode on average 12 BGCs, which is over twice the average of five BGCs generally found in cyanobacteria, although some genomes can have more than 20 BCGs [23]. This richness of natural product BGC is consistent with the diversity of toxins and other natural products reported from this group of cyanobacteria. As a result of versatile BGCs, analysis of genomic datasets from cyanobacteria can lead to the study of hundreds of diverse gene clusters [55]. Despite the extensive work, accessing these vast pathways may provide new insights into the biosynthetic repertoire and even the evolutionary history of cyanobacteria can be obtained [24,56,57]. Computational analysis and comparison of gene clusters can thus facilitate organizing this vast biosynthetic diversity and help explore cryptic BGCs [58].

The network generated with the automatic annotation of regions with BGCs in the 31 ADA clade genomes provided highlighted patterns in the natural products distributions. The antifungal hassallidin was identified only in planktonic *Dolichospermum* strains from Finland, even though this molecule is considerably widespread in other cyanobacterial genera [59]. Finnish *Dolichospermum* and *Anabaena* (*Dolichospermum*) spp. LE011-2 and AL09 (regions not presented in the network due to being singletons), which all encode gas vesicle gene clusters, were the only strains to present the anabaenopeptilide gene cluster. Geosmin, a terpenoid that causes an earthy smell and taste in water, is encoded in Australian *Dolichospermum* and *Anabaena* (*Dolichospermum*) [60]. *Dolichospermum* from Finland and *Anabaena* (*Dolichospermum*) from the USA presented the gene cluster of the antimicrobial cyanobactin anacyclamide [61]. Nonetheless, the majority of the clusters in the networks could not be associated with known natural products and remains to be identified by future research with the ADA clade. Many cyanobacterial natural products are potential drug leads [23] and therefore the BGC-rich *Anabaena/Dolichospermum/Aphanizomenon* genera complex remains a significant study subject.

## 4. Conclusions

We sequenced 11 new draft genomes that cluster in the ADA clade and screened all available genomes of *Anabaena*, *Dolichospermum* and *Aphanizomenon* for biosynthetic gene clusters. The genomes presented here established new subgroups in the ADA clade (ADA-γ, ADA-ζ, and ADA-ε) and expanded the known biosynthetic diversity of the three cyanobacterial genera. Thus far, biosynthetic gene cluster distribution follows phylogenomic clustering within the ADA clade, as strains that produce microcystin, anatoxin-a, and saxitoxin belong to three specific subclades. The phylogenetic separation of benthic *Anabaena* and planktonic *Dolichospermum* is less clear than previously thought. Future studies with the clade should focus on expanding the genomic information of representative strains from diverse geographical locations and benthic strains to maximize phylogenetic coverage.

## 5. Materials and Methods 

### 5.1. Strains and Toxin Detection

All UHCC strains in this study belong to the University of Helsinki culture collection HAMBI. The strains were from environmental samples and were maintained in collection in liquid Z8 medium (UHCC 0167, 0299 and 0966) or Z8X medium (Z8 without nitrogen, UHCC 0187, 0204, 0253, 0037, 0259, 0260, 0352, 0406, and 0183) in constant light (3–15 µmolm^−2^ s^−1^) at 20 to 25 °C [62]. 

The microcystin production of *Anabaena* sp. UHCC 0352 was detected with LC-MS from freeze-dried sample. Toxins were extracted with 70% ethanol and samples were injected into an Acquity UPLC system (Waters, Manchester, UK), equipped with a Kinetex^®^ 1.7 µm C8 100 Å, LC Column 50 × 2.1 mm. The UPLC was operated with a flow-rate of 0.3 mL/min in gradient mode, at a temperature of 40 °C. Solvents used in the gradient were A: 0.1% formic acid in water and B: 0.1% formic acid in acetonitrile/isopropanol (1:1). The initial conditions of the linear gradient were A: 95% and B: 5% and the ratio was changed to A: 0% and B: 100% in 5 min and stayed there 2 min. The ratio was changed to A: 95% and B: 5% in 0.5 min and stayed there for 2.5 min. Injection volume was 1.0 μL. Mass spectra were recorded with a Waters Synapt G2-Si mass spectrometer (Waters, Manchester, UK). Measurements were performed using positive electrospray ionization (ESI) in resolution mode. Ions were scanned in the range from m/z 50 to 2000. MS analyses were performed with scan times of 0.1 s. Capillary voltage was 1.5 kV, source temperature 120 °C, sampling cone 40.0, source offset 80.0, desolvation temperature 600 °C, desolvation gas flow 1000 L/h and nebulizer gas flow 6.5 Bar. Leucine-encephalin was used as a lock mass (10s interval) and calibration was done with sodium formiate and Ultramark 1621. Trap Collision Energy Ramp of the high energy channel started from 20.0 eV and ended to 40.0 eV. UV-VIS was monitored in the range from 210 to 800 nm.

### 5.2. Genome Sequencing and Assembly

Genomic DNA of the 11 UHCC strains was extracted according to previously described methods [63]. Isolated DNA concentrations were measured using a NanoDrop 1000 spectrophotometer (Thermo Scientific, Waltham, MA, USA) and quality with an Agilent TapeStation (Agilent Technologies, Santa Clara, CA, USA). 

DNA of UHCC 0183, 0187, 0204, 0253, and 0260 was used for library preparation construction (Illumina TruSeq^®^ PCR Free 350bp, Illumina, San Diego, CA, USA) and sequenced by an Illumina HiSeq2500 (Illumina, San Diego, CA, USA) platform with a paired-ends 100-cycle run. For UHCC 0167, 0259, 0299, 0352, and 0406, genomic DNA was sheared, end-repaired, and size selected using a 0.6% agarose gel before 3Kb-insert library construction. Sequencing was conducted by an Illumina HiSeq2000 (Illumina) platform with a paired-ends 100-cycle run. Genomic DNA of UHCC 0037 was sequenced using a Roche 454 Genome Sequencer (GS) FLX (F. Hoffmann-La Roche, Basel, Switzerland) and Illumina Genome Analyzer II (GA IIx) (Illumina).

The programs PRINSEQ v0.20.4 [64] and Trimmomatic v0.33 [65] were used for quality control and adapter removal, respectively. The threated reads were corrected by Spades 3.7.1 [66]. Genome assembly of UHCC 0037 was conducted with Newbler 2.0.0 [67] while the assemblies of the other 10 strains were conducted using Newbler 3.0 [67]. The assemblies were followed by scaffolding and gap closure by Platanus 1.2.4 [68].

Post-assembly taxonomic assignment was performed using Kraken v2 [69] and ZEUSS [70]. The program CheckM v1.1.2 was used for calculating the completeness and redundancy [71] (10.1101/gr.186072.114). Other genome statistics were obtained using Assemblathon 2 [72].

### 5.3. Phylogenetic, Phylogenomic, and Comparative Genomics

A maximum-likelihood phylogenomic tree was constructed by RAxML v.8.2.12 [73] using an alignment of 31 universal marker genes [24,25] from 94 cyanobacterial genomes with 1000 rapid bootstrap searches. Protein sequences were obtained using [74] collection in anvi’o v5.5 workflow for phylogenomics [75]. The model PROTGAMMAIGTR was assigned as the best by ProtTest 3.4.2 [76]. The 16S rRNA inference with a total of 109 sequences from cyanobacterial genomes was conducted based on the nucleotide substitution model HKY+I+G assigned by a BIC calculation in jModelTest v2.1.10 [77] and 5,000,000 generations in the program MrBayes [78]. MEGA v10.0.5 (default parameters for nucleotides and amino acids) was used for the alignment of both conserved proteins and 16S rRNA sequences [79].

The 16S rRNA sequence identity comparison was performed using the default parameters on SIAS (http://imed.med.ucm.es/Tools/sias.html). Possible contaminants were excluded (*Anabaena* sp. MDT14b 16S rRNA) and *Anabaena* spp. AL09, LE011-02, WA93, UBA12330, and CRKS33 genome assemblies did not contain the 16S RNA gene. Pan- and core-genome estimations of 30 ADA genomes using the NCBI prokaryotic genome annotation pipelines in Genbank were made with the OrthoMCL algorithm and Tettelin function in the program GET_HOMOLOGUES v3.2.2 [80,81]. The strain UKL13-PB was not included here due to the absence of public PGAP annotation. The program was also employed alongside a seaborn v0.9 library heatmap script [82] for generating the average amino acid and nucleotide identity heatmaps.

### 5.4. BGC and Gas Vesicle Gene Cluster Annotation

The 31 genomes analyzed in the present work were screened for BGCs using antiSMASH 5.0 [83]. Genes in intact BGCs were given a proposed function as their syntenic counterparts in similar clusters using BLASTp [84] searches. Regions associated with natural products identified by antiSMASH 5.0 were organized in a network using BiG-SCAPE [58] and Cytoscape 3.7.1 [85]. Inkscape [86] was used for figure editing. Gas vesicle gene clusters were screened with BLASTp using *Dolichospermum* sp. UHCC 0315 gas vesicle genes as a query [19]. 

### 5.5. Data Availability

Genome assemblies of 11 newly sequenced strains were deposited in NCBI under the BioProject accession numbers PRJNA549399 (*Dolichospermum flos-aquae* UHCC 0037), PRJNA549403 (*Dolichospermum planctonicum* UHCC 0167), PRJNA549389 (*Aphanizomenon* sp. UHCC 0183), PRJNA549386 (*Anabaena* sp. UHCC 0187), PRJNA549375 (*Anabaena* sp. UHCC 0204), PRJNA549393 (*Anabaena* sp. UHCC 0253), PRJNA549376 (*Dolichospermum* sp. UHCC 0259), PRJNA549379 (*Dolichospermum* sp. UHCC 0260), PRJNA549373 (*Dolichospermum* sp. UHCC 0299), PRJNA549377 (*Dolichospermum* sp. UHCC 0352), PRJNA549385 (*Dolichospermum* sp. UHCC 0406).

## Supplementary Materials

The following are available online at https://www.mdpi.com/2072-6651/12/4/248/s1, Figure S1: Bayesian inference tree based on 16S rRNA gene (**a**); and average nucleotide (**b**) and amino acid (**c**) identity heatmaps of the *Anabaena/Dolichospermum/Aphanizomenon* (ADA) clade. Genomes investigated in the present study are shown in bold. Known toxin producers are indicated with colored dots [7,9,10,26,29,30]. Color coding for ADA-subgroups and toxins is the same as in Figure 1. Sequence accession numbers are within parentheses. The heatmaps were generated using genomes included in this work and with public automatic annotation deposited in GenBank, Figure S2: Maximum likelihood tree constructed using 31 universal marker genes [24,25] encoded in 94 cyanobacterial genomes from diverse cyanobacterial families. The proposed *Anabaena/Dolichospermum/Aphanizomenon* clade subgroups [6,19] and natural products biosynthetic gene clusters are indicated by different colors. Genome accession numbers in NCBI are presented within parentheses, Figure S3: Representation of the biosynthetic gene clusters identified through manual curation in the 31 analyzed *Anabaena/Dolichospermum/Aphanizomenon* genomes. See also Table S2, Figure S4: Representation of the gas vesicle clusters identified through manual curation in the 31 analyzed *Anabaena*/*Dolichospermum*/*Aphanizomenon* genomes. See also Table S2, Figure S5: Core- (**a**) and Pan- (**b**) genome estimations of 30 analyzed *Anabaena/Dolichospermum/Aphanizomenon* genomes that have automatic annotations generated in the GenBank database. The strain UKL13-PB was not included here due to the absence of public PGAP annotation, Table S1: Genomic features of the 31 *Anabaena/Dolichospermum/Aphanizomenon* genomes included in the present work, Table S2: Proposed function of proteins encoded in the biosynthetic gene clusters identified in the *Anabaena/Dolichospermum/Aphanizomenon* genomes evaluated in this study, Table S3: 16S rRNA gene sequence similarity matrix (p-distance) of the *Anabaena/Dolichospermum/Aphanizomenon* clade genomes, Table S4: Average nucleotide identity values for genome pairs using GET_HOMOLOGUES software. Comparison between previous results with ANIcalculation program [6] and GET_HOMOLOGUES highlighted in blue.
