# Supplementary figures and images for: Phylogenomic Analysis of Secondary Metabolism in the Toxic Cyanobacterial Genera Anabaena, Dolichospermum and Aphanizomenon
Source: Toxins (Basel). 2020 Apr 11;12(4):248. doi: 10.3390/toxins12040248 (PMC7232259; doi:10.3390/toxins12040248)

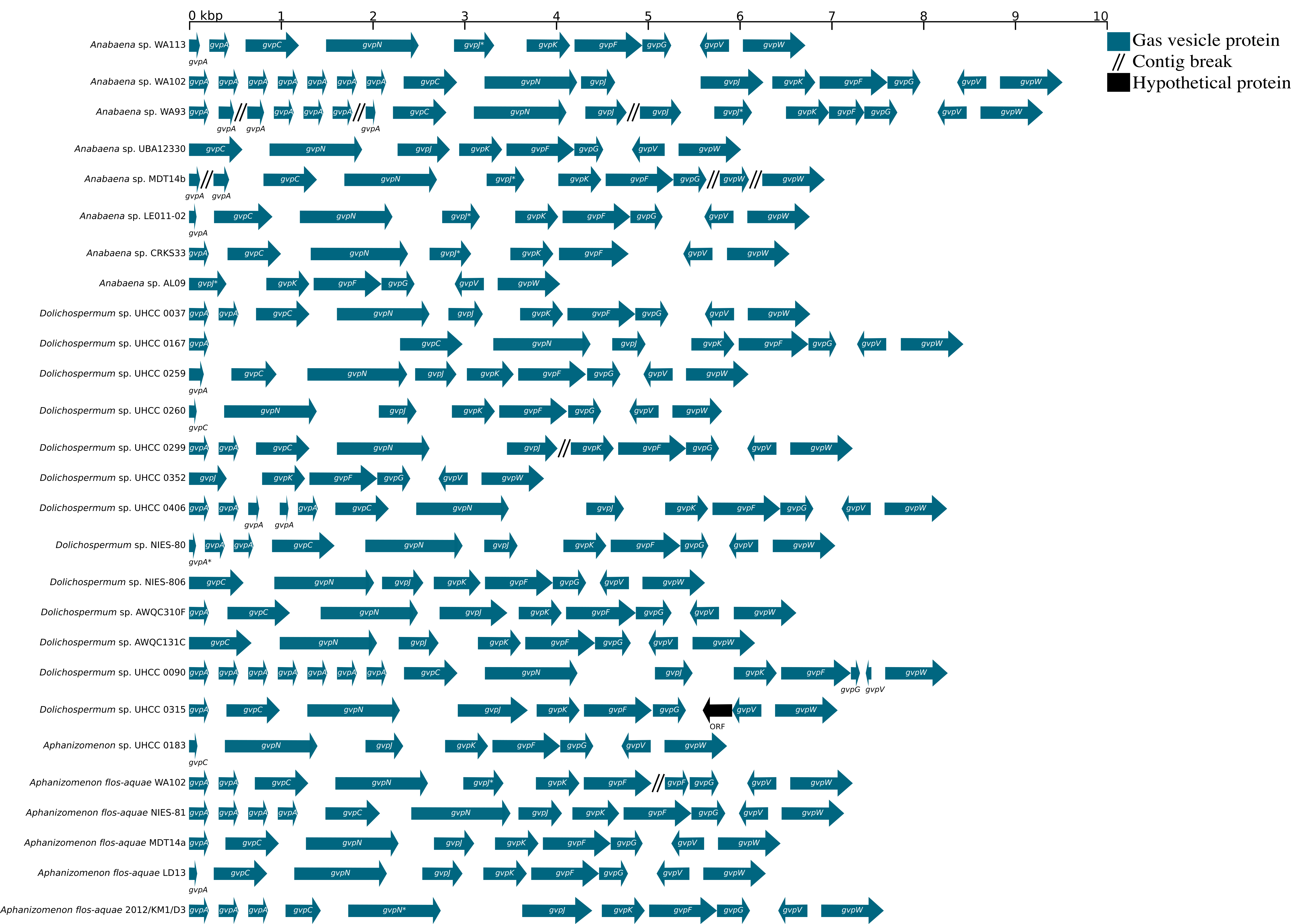

Supplement: Supplementary file 1 [file toxins-12-00248-s001.zip › Toxins-739260_suplementary-final ╢■┤╬proof/FigS4(gvp_cluster).tiff]

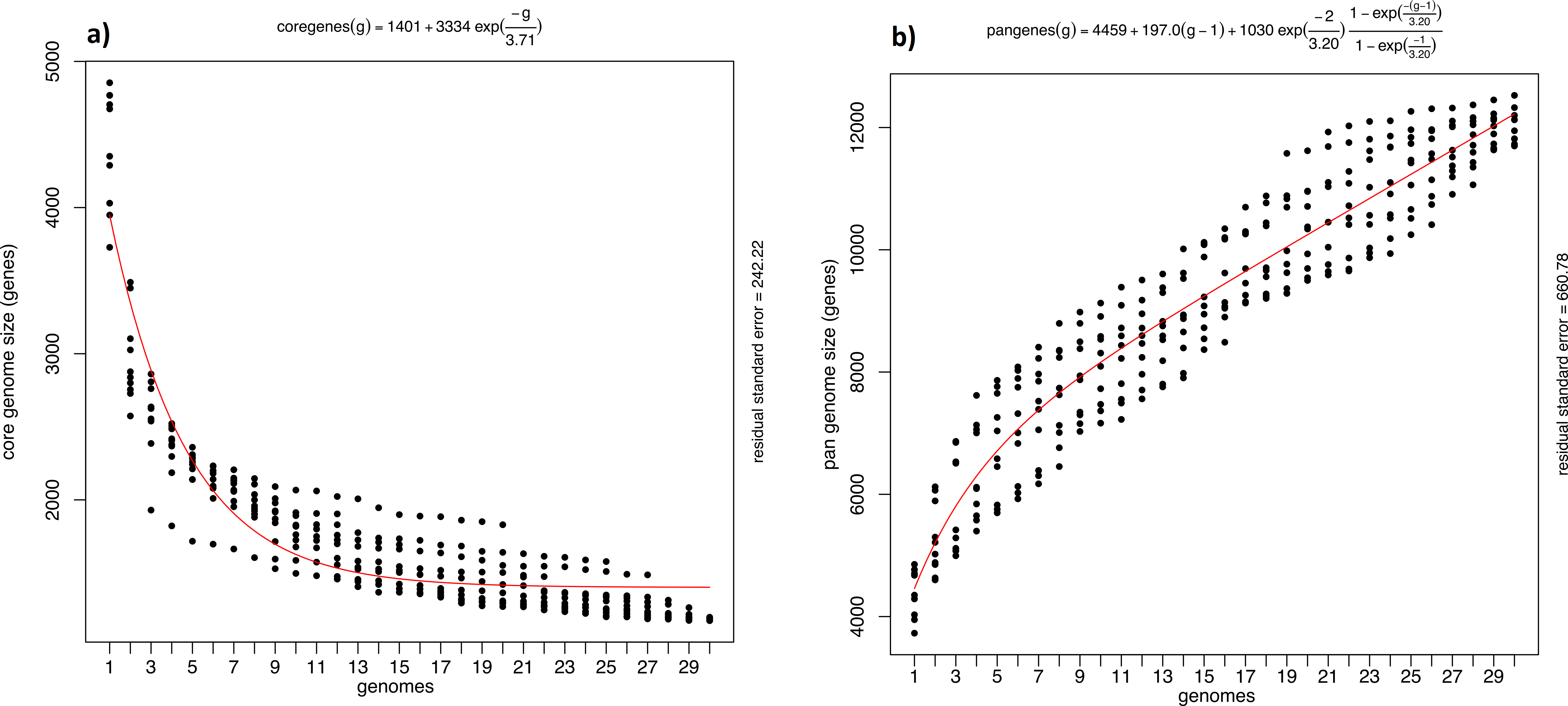

Supplement: Supplementary file 1 [file toxins-12-00248-s001.zip › Toxins-739260_suplementary-final ╢■┤╬proof/FigS5(pan_core).tif]
